# Inflammatory subphenotypes previously identified in ARDS are associated with mortality at intensive care unit discharge: a secondary analysis of a prospective observational study

**DOI:** 10.1186/s13054-024-04929-9

**Published:** 2024-05-07

**Authors:** Marleen A. Slim, Rombout B. E. van Amstel, Lieuwe D. J. Bos, Olaf L. Cremer, MARS Consortium, Friso M. de Beer, Lieuwe D. J. Bos, Gerie J. Glas, Arie J. Hoogendijk, Roosmarijn T. M. van Hooijdonk, Janneke Horn, Mischa A. Huson, Laura R. A. Schouten, Marcus J. Schultz, Brendon P. Scicluna, Marleen Straat, Lonneke A. van Vught, Luuk Wieske, Maryse A. Wiewel, Esther Witteveen. Marc J. M. Bonten, Olaf M. Cremer, David S. Y. Ong, Jos F. Frencken, Peter M. C. Klein Klouwenberg, Maria E. Koster‐Brouwer, Kirsten van de Groep, Diana M. Verboom, W. Joost Wiersinga, Tom van der Poll, Lonneke A. van Vught

**Affiliations:** 1 Center for Experimental and Molecular Medicine, Amsterdam University Medical Center, Amsterdam Institute for Infection and Immunity, University of Amsterdam, Meibergdreef 9, 1105 AZ Amsterdam, The Netherlands; 2 Department of Intensive Care, Amsterdam University Medical Center, Amsterdam Institute for Infection and Immunity, University of Amsterdam, Amsterdam, The Netherlands; 3 Department of Intensive Care Medicine, University Medical Center Utrecht, Utrecht, The Netherlands; 4 Department of Medicine, Division of Infectious Diseases, Amsterdam University Medical Center, University of Amsterdam, Amsterdam, The Netherlands

**Keywords:** Intensive care, Subphenotypes, Mortality, Biomarkers, Coagulation, Endothelium, Inflammation

## Abstract

**Background:**

Intensive care unit (ICU)-survivors have an increased risk of mortality after discharge compared to the general population. On ICU admission subphenotypes based on the plasma biomarker levels of interleukin-8, protein C and bicarbonate have been identified in patients admitted with acute respiratory distress syndrome (ARDS) that are prognostic of outcome and predictive of treatment response. We hypothesized that if these inflammatory subphenotypes previously identified among ARDS patients are assigned at ICU discharge in a more general critically ill population, they are associated with short- and long-term outcome.

**Methods:**

A secondary analysis of a prospective observational cohort study conducted in two Dutch ICUs between 2011 and 2014 was performed. All patients discharged alive from the ICU were at ICU discharge adjudicated to the previously identified inflammatory subphenotypes applying a validated parsimonious model using variables measured median 10.6 h [IQR, 8.0–31.4] prior to ICU discharge. Subphenotype distribution at ICU discharge, clinical characteristics and outcomes were analyzed. As a sensitivity analysis, a latent class analysis (LCA) was executed for subphenotype identification based on plasma protein biomarkers at ICU discharge reflective of coagulation activation, endothelial cell activation and inflammation. Concordance between the subphenotyping strategies was studied.

**Results:**

Of the 8332 patients included in the original cohort, 1483 ICU-survivors had plasma biomarkers available and could be assigned to the inflammatory subphenotypes. At ICU discharge 6% (n = 86) was assigned to the hyperinflammatory and 94% (n = 1397) to the hypoinflammatory subphenotype. Patients assigned to the hyperinflammatory subphenotype were discharged with signs of more severe organ dysfunction (SOFA scores 7 [IQR 5–9] vs. 4 [IQR 2–6], *p* < 0.001). Mortality was higher in patients assigned to the hyperinflammatory subphenotype (30-day mortality 21% vs. 11%, *p* = 0.005; one-year mortality 48% vs. 28%, *p* < 0.001). LCA deemed 2 subphenotypes most suitable. ICU-survivors from class 1 had significantly higher mortality compared to class 2. Patients belonging to the hyperinflammatory subphenotype were mainly in class 1.

**Conclusions:**

Patients assigned to the hyperinflammatory subphenotype at ICU discharge showed significantly stronger anomalies in coagulation activation, endothelial cell activation and inflammation pathways implicated in the pathogenesis of critical disease and increased mortality until one-year follow up.

**Supplementary Information:**

The online version contains supplementary material available at 10.1186/s13054-024-04929-9.

## Background

Critically ill patients who survive the acute phase of their disease show persistent cognitive, physical and functional impairment, leading to lower quality of life, increased readmission rates and an enhanced risk of mortality [1]. Intensive care unit (ICU) survivors use more healthcare resources after hospital discharge compared to all hospitalized patients, and one-third of ICU patients discharged alive die within 5 years after discharge [2]. During and after ICU admission, especially for sepsis, profound and persistent alteration of the immune response in survivors has been observed [3] and this sustained dysregulation of the host immune response after hospital discharge is hypothesized to contribute to long-term sequelae [4]. Worsened physical, mental and neurocognitive status in ICU-survivors is called post-intensive care syndrome (PICS), and is not only related to the disease severity during ICU admission [1], calling for a more personalized approach.

Substantial progress has been made in the identification of subphenotypes of critical care syndromes, which have been prognostic of outcome and predictive of treatment response [5]. Subphenotypes have been identified for acute respiratory distress syndrome (ARDS) [6, 7], sepsis [8–10] and acute kidney injury (AKI) [11, 12], resulting in new clinical, biological and predictive insights. ARDS patients have been divided in a hyperinflammatory and a hypoinflammatory subphenotype using a latent class analysis (LCA) with clinical and plasma protein biomarker data [6]. These subphenotypes were identified within 36 h after the diagnosis of ARDS, and were found to respond differently to several treatments including administration of simvastatin, fluid management and positive and-expiratory pressure (PEEP) mechanical ventilation strategy in retrospective analyses [6, 13, 14]. These subphenotypes might not be disease specific; extending these ARDS subphenotypes to mechanically ventilated critically ill patients without ARDS [15] and in patients with sepsis [16] showed similar results, suggesting that these subphenotypes might be a universal predictor for outcome and treatment response in critically ill patients.

To date, only scarce data exist on patient stratification at ICU discharge. Whether these subphenotypes show association with short or long-term outcome is yet to be determined. We hypothesized that if these inflammatory subphenotypes previously identified among ARDS patients are assigned at ICU discharge, they are associated with worse clinical short and long-term outcomes and a more dysregulated host response. The present study seeks to identify subphenotypes in a more broadly defined population of critically ill ICU-survivors, consisting of sepsis, ARDS and non-infectious patients, at ICU discharge and investigates the association of these subphenotypes with one-year mortality.

## Methods

### Study design and patient selection

This study was conducted as a secondary analysis of the Molecular Diagnosis and Risk Stratification of Sepsis (MARS) cohort, a prospective observational cohort study in the mixed ICUs of 2 tertiary teaching hospitals (Amsterdam Universal Medical Centers, location AMC, in Amsterdam and University Medical Center Utrecht in Utrecht) [17]. The MARS study included all patients admitted to the ICU above 18 years of age between January 2011 and January 2014 with an expected length of stay longer than 24 h. Patients were included via an opt-out method approved by the medical ethical committees of the participating hospitals. Information on demographics, comorbidities, daily clinical, laboratory and outcome data were prospectively collected by trained and dedicated ICU researchers [18]. The diagnosis on admission was also collected. Patients were either classified as having sepsis or a noninfectious admission diagnosis. Sepsis was defined as the presence of an infection diagnosed within 24 h after ICU admission with a probable or definite likelihood [19] accompanied by a Sequential Organ Failure Assessment (SOFA) scores ≥ 2 according to the sepsis-3 criteria [20]. In patients with a noninfectious admission diagnosis (including those admitted with suspected infection but with a post hoc infection likelihood of none), the Acute Physiology and Chronic Health Evaluation IV (APACHE IV) primary admission diagnosis was used to classify patients [21]. In a retrospectively selected subset of the patients admitted between January 2011 and July 2013 plasma protein biomarkers were measured. This subset consisted of patients with an infection with a probable or definite likelihood, patients with ARDS or patients selected as non-infectious ICU controls [17, 22, 23] (infection likelihood criteria are described in [18], ARDS criteria in [24]). For more information of the selection of this subset see the Additional file 1: Supplementary methods. Patients were selected (1) who were discharged alive from the ICU, and (2) of whom plasma protein biomarkers were available and collected on the day of ICU discharge, or one or two day(s) before. ICU readmissions within the same hospital admission were excluded from the analyses. However, ICU readmissions were collected as outcome measurements reported in two ways; ICU readmissions within the same hospital admission and all ICU readmissions during the remaining study duration of MARS. Baseline characteristics, comorbidities, outcome data and severity indices such as APACHE IV [21] and SOFA scores [20] are reported. For information on the definitions of comorbidities and complications, see the Additional file 1: Supplementary methods.

### Biomarker measurements

Host response biomarkers reflective of coagulation-, endothelial and inflammatory pathways were measured in ethylenediaminetetraacetic acid (EDTA) anticoagulated plasma, collected within 16 h after ICU admission, on day 2, on day 4 and on ICU discharge (-1 or -2 days). Biomarker panels consisted of angiopoietin-1, angiopoietin-2, D-dimer, interferon-γ, interleukin (IL)-6, IL-8, IL-10, fractalkine, matrix metalloproteinase (MMP)-8, protein C, soluble E-selectin and soluble intercellular adhesion molecule-1 (ICAM-1). C-reactive protein (CRP), creatinine lactate, platelets, prothrombin time (PT), bicarbonate and white blood cell count (WBC), amongst others, were measured for clinical purposes. For further information on sample handling and assays, see the Additional file 1: Supplementary methods.

### Adjudication to subphenotypes

The probability of belonging to the hyperinflammatory or hypoinflammatory subphenotype [6] was estimated using a previously published and validated parsimonious 3-variable model using plasma levels of bicarbonate, IL-8 and Protein C at ICU discharge [25]. To assure a reliable subphenotype adjudication plasma bicarbonate results were aligned with the day of IL-8 and Protein C measurements. When the bicarbonate value was missing on the day of the IL-8 and protein C measurement, (1) the average of the bicarbonate value on the day before and the day after was used, or (2) if there was only one value available (on the day before or after), this value was carried forward or backward, or (3) when both values on the day before and after were missing, the patient was excluded. A probability of > 0.5 was used to assign patients to the hyperinflammatory subphenotype, and < 0.5 to the hypoinflammatory subphenotype.

### Outcome measurements

The study aim was to analyze associations between the biological subphenotypes and the primary and secondary outcomes. The primary outcomes were short- and long-term mortality (30-day, 90-day mortality and one-year mortality) after ICU discharge. The secondary outcomes were ICU readmission after ICU discharge and biomarkers reflective of the coagulation-, endothelial and inflammatory pathways were compared between the different subphenotypes at ICU discharge.

### Statistical analysis

Patient characteristics, plasma protein biomarker concentrations, and outcomes were compared using a t-test or one-way ANOVA for parametric data, a Mann–Whitney U test or Kruskal–Wallis test for nonparametric data, and with Chi-square test for categorical data, stratified by subphenotype. Subphenotype consistency was analyzed by adjudication of subphenotypes on ICU admission and readmission and assessing subphenotype overlap. The percentage of the patients in the subphenotypes was compared at different time points (ICU admission vs. ICU discharge, ICU discharge vs. ICU readmission), visualized with mosaic plots. Differences in individual plasma protein biomarker levels between the previously described subphenotypes were quantified and expressed as Hedges’ g, a commonly used effect size measure [26]. Survival was visualized using Kaplan–Meier curves and analyzed using a Cox proportional hazards model. Previously identified risk factors for long-term outcome after ICU survival were included in the Cox regression models [27]. These risks factors consisted of age, Charlson comorbidity index [28], chronic cardiovascular insufficiency, chronic heart failure, chronic kidney disease, malignancy, systolic blood pressure, temperature, platelets, white blood cell count and length of ICU stay at ICU discharge. As a sensitivity analysis to investigate the influence of admission diagnoses and complications, the adjusted Cox regression analysis was repeated with the following factors also included: complications upon ICU admission (ARDS, sepsis and AKI), admission diagnosis group (cardiovascular, gastrointestinal, metabolic, neurological, other, respiratory and trauma) and admission type (medical or surgical). A *p* value of < 0.05 was considered of statistical significance.

A sensitivity analysis was conducted using a LCA based on plasma protein biomarker concentrations in the coagulation, endothelial and inflammatory pathways (plasma protein biomarkers that were not part of the daily clinical laboratory measurements) at ICU discharge to identify classes of ICU survivors. The previously identified subphenotypes in ARDS patients were based on clinical and biological data, for this sensitivity analysis we aimed to analyze if similar patterns would emerge by only using non-daily clinical laboratory measurements. The process of model design and LCA followed the steps and considerations outlined by Sinha et al. [29]. First, missing plasma protein biomarkers used in the LCA were imputed using multivariate imputation by chained equations algorithm (see the Additional file 1: Supplementary methods) and transformed to resemble normally distributed data, which was verified using Shapiro–Wilk tests and histograms. If plasma protein biomarkers had a Spearman correlation coefficient > 0.6, one of the two was excluded from the LCA. Plasma biomarker levels were scaled by subtracting the mean in this cohort and dividing by the standard deviation. Five sequential models consisting of 1 to 5 classes were fit. As described [29] the best-fitting model was selected based on the Bayesian information criterion, the Vuong-Lo-Mendell-Rubin test [30] and entropy. Subphenotype characteristics were visualized using a profile plot, which displayed the mean standardized differences of subphenotype defining variables. To conduct LCA Mplus was used. Once the best model was selected, an individual patient’s class assignment was determined by the highest probability of class membership. The percentage of the patients in the subphenotypes was compared between the two clustering strategies at ICU discharge (adjudication of previously described subphenotypes vs. LCA), visualized with a mosaic plot.

## Results

### Population

**Fig. 1:**
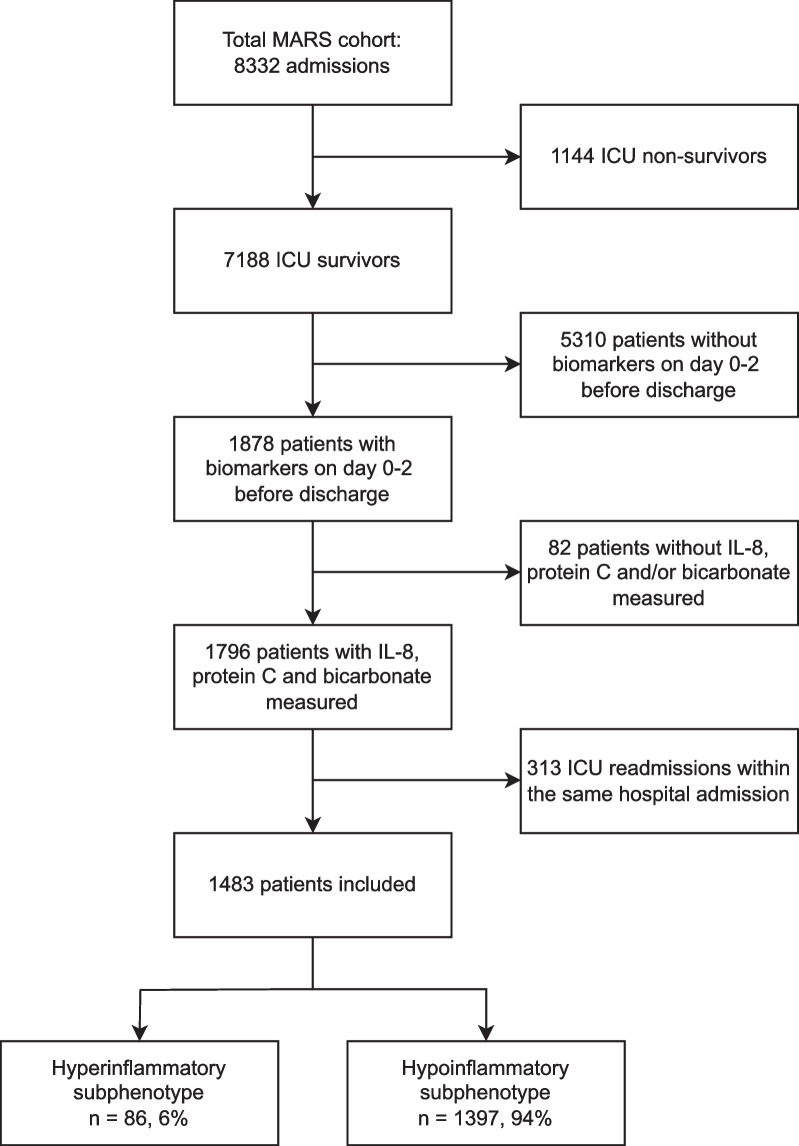
Flowchart of patient selection. *ICU* intensive care unit; *IL* interleukin

Of the 8332 patients included in the original cohort, 1483 ICU survivors were selected for further subphenotyping (Fig. 1). The included patients had a median age of 62 [IQR 50–71] years and 61% was male (n = 907) (Table 1). On ICU admission, 292 (20%) patients had ARDS and 927 (63%) sepsis, of whom 257 (17%) had septic shock and 252 (17%) had both sepsis and ARDS (Table 1). The most common comorbidities were cardiovascular compromise (n = 440, 30%), hypertension (n = 438, 30%), immunocompromise (n = 295, 20%) and diabetes (n = 288, 19%) (Table 1). For the most common diagnoses on admission see Additional file 1: Supplementary Table 1. The APACHE IV score and the SOFA score upon ICU admission were 70 [IQR 54–90] and 6 [IQR 4–8] respectively.

**Table 1:** Characteristics at ICU admission of ICU survivors, stratified at ICU discharge according to previously defined inflammatory subphenotypes

	Hyperinflammatory n = 86, 6%	Hypoinflammatory n = 1397, 94%	*p*-value
*Demographics*			
Age (median [IQR])	64 [51–71]	62 [50–71]	0.973
Sex = male (%)	38 (44.2)	869 (62.2)	0.001
Race = White (%)	71 (82.6)	1208 (86.5)	0.389
*Medical history*			
No comorbidities (%)	25 (29.1)	408 (29.2)	> 0.999
Cardiovascular compromise (%)	15 (17.4)	425 (30.4)	0.015
COPD (%)	4 (4.7)	172 (12.3)	0.050
Diabetes (%)	11 (12.8)	277 (19.8)	0.144
Hypertension (%)	26 (30.2)	412 (29.5)	0.981
Immunocompromise (%)	26 (30.2)	269 (19.3)	0.020
Malignancy (%)	20 (23.3)	224 (16.0)	0.109
Renal insufficiency (%)	11 (12.8)	149 (10.7)	0.662
Respiratory insufficiency (%)	5 (5.8)	234 (16.8)	0.012
Charlson comorbidity score (median [IQR])	3 [2–4]	3 [1–5]	0.939
*Admission type and diagnosis*			
Surgical admission (%)	7 (8.1)	281 (20.1)	0.010
Diagnosis on admission* (%)			< 0.001
Cardiovascular	12 (14.0)	311 (22.3)	
Gastrointestinal	37 (43.0)	245 (17.5)	
Metabolic	1 (1.2)	13 (0.9)	
Neurological	1 (1.2)	99 (7.1)	
Other	19 (22.1)	187 (13.4)	
Respiratory	15 (17.4)	499 (35.7)	
Trauma	1 (1.2)	43 (3.1)	
*On admission (day 0 & 1)*			
Mechanical ventilation (%)	45 (52.3)	1143 (81.8)	< 0.001
Shock (%)	19 (22.1)	360 (25.8)	0.525
Acute kidney injury (%)	40 (46.5)	400 (28.6)	0.001
ARDS (%)	6 (7.0)	286 (20.5)	0.004
Sepsis (%)	68 (79.1)	859 (61.5)	0.002
Of which septic shock	16 (18.6)	241 (17.3)	0.863
Of which also ARDS	6 (7.0)	246 (17.6)e	0.016
*Disease severity on admission*			
APACHE IV Score (median [IQR])	73 [61–95]	70 [53–89]	0.044
SOFA (median [IQR])	7 [5–9]	6 [4–8]	0.027

*APACHE* acute physiology and chronic health evaluation; *ARDS* acute respiratory distress syndrome; *COPD* chronic obstructive pulmonary disease; *SOFA* sequential organ failure assessment

*Diagnoses on admission are based on the APACHE IV admission diagnoses. See Additional file 1: Supplementary Table 1 for the most common diagnoses per diagnosis group

### Inflammatory subphenotypes previously identified among ARDS patients

In 959 (65%) of the included patients plasma biomarker levels were measured on the day of ICU discharge, in 419 (28%) the day before ICU discharge and in 105 (7%) patients 2 days before ICU discharge; this was approximately equally distributed in both subphenotypes (Additional file 1: Supplementary Table 2). For 307 (21%) this entailed that the plasma biomarker levels used for this analysis were taken upon ICU admission since no later sample was available. At ICU discharge the majority of patients (n = 1397, 94%) were adjudicated to the hypoinflammatory subphenotype whereas the hyperinflammatory subphenotype consisted of 86 patients (6%) (Fig. 1). Patients adjudicated to the hyperinflammatory subphenotype were discharged with more severe disease reflected by higher SOFA scores (7 [IQR 5–9] vs. 4 [IQR 2–6], *p* < 0.001). The number of ICU-acquired complications (shock, AKI, ARDS and sepsis) that had occurred prior to discharge was comparable between the two subphenotypes (Table 2). Patients within the hyperinflammatory subphenotype at discharge had been more severely ill upon admission (APACHE IV score 73 vs. 70, *p* = 0.044 and SOFA score 7 vs. 6, *p* = 0.027), were more often admitted with sepsis (79% vs. 62%, *p* = 0.002) and less often admitted with ARDS (7% vs. 21%, *p* = 0.004) (Table 1).

**Table 2:** Characteristics and outcome at ICU discharge of ICU survivors, stratified at ICU discharge according to previously defined inflammatory subphenotypes

	Hyperinflammatory n = 86, 6%	Hypoinflammatory n = 1397, 94%	*p*-value
*Disease severity at ICU discharge*			
SOFA score (median [IQR])	7 [5–9]	4 [2–6]	< 0.001
*ICU acquired (*≥ *day 2)*			
Shock (%)	3 (3.5)	61 (4.4)	0.907
Acute kidney injury (%)	4 (4.7)	96 (6.9)	0.565
ARDS (%)	0 (0.0)	63 (18.1)	0.543
Sepsis (%)	5 (5.8)	133 (9.5)	0.335
*Restrictions at ICU discharge*			
Restrictions on care (%)			0.249
No restrictions	76 (88.4)	1227 (88.0)	
Do not resuscitate	2 (2.3)	85 (6.1)	
Extended restrictions	6 (7.0)	70 (5.0)	
Palliative care	2 (2.3)	13 (0.9)	
*Outcomes*			
LOS hospital (median [IQR])	16.5 [9–43]	16.5 [9–33]	0.359
LOS ICU (median [IQR])	2 [1–3]	4 [2–7]	< 0.001
Hospital mortality (%)	17 (19.8)	160 (11.5)	0.033
Days until mortality (median [IQR])	37 [13–134]	48 [12–144]	0.579
30-day mortality (%)	18 (21.4)	149 (10.8)	0.005
90-day mortality (%)	28 (33.3)	236 (17.3)	< 0.001
1-year mortality (%)	40 (47.6)	378 (27.7)	< 0.001
ICU readmission within the same hospital admission (%)	21 (24.4)	222 (15.9)	0.054
ICU readmission in total (%)*	31 (36.0)	315 (22.5)	0.006

*ICU readmissions during the complete duration of the MARS study between January 2011 and January 2014

*ARDS* acute respiratory distress syndrome; *ICU* intensive care unit; *LOS* length of stay; *SOFA* sequential organ failure assessment

### Subphenotype overlap at ICU discharge versus ICU admission

**Fig. 2:**
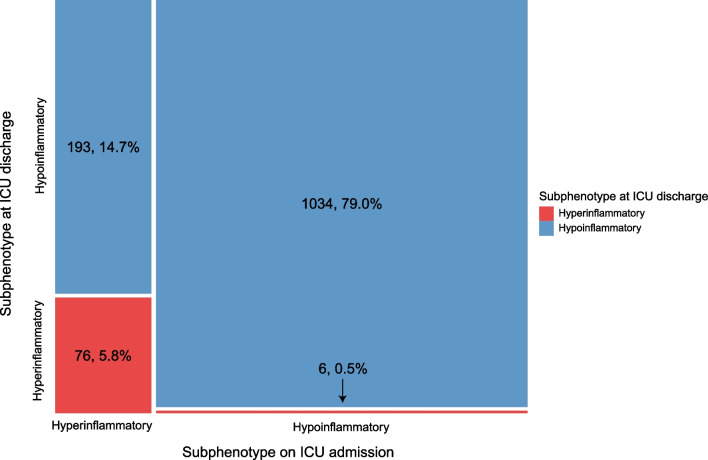
Mosaic plot showing allocation of inflammatory subphenotypes on ICU admission and at ICU discharge. Mosaic plot showing the allocation of the inflammatory subphenotypes on ICU admission and at ICU discharge of the 1309 (88%) patients in this cohort of which the subphenotypes on admission was known. Patients with the hyperinflammatory subphenotype at ICU discharge are depicted in red, with the hypoinflammatory subphenotype in blue. In the left upper corner the patients are displayed who were hypoinflammatory at ICU discharge and hyperinflammatory on ICU admission (n = 193, 14.7%), in the left lower corner the patients who were both hyperinflammatory at ICU discharge and on ICU admission (= 76, 5.8%). In the right upper corner the patients are displayed who were both hypoinflammatory at ICU discharge and on ICU admission (n = 1034, 79.0%), in the right lower corner the patients who were hyperinflammatory at ICU discharge and hypoinflammatory on ICU admission (n = 6, 0.5%%). In 307 (23%) of the patients, the adjudication to the subphenotypes both at ICU admission and ICU discharge was based on the same plasma protein biomarker measurements. *ICU* intensive care unit

Of 1309 patients (88%) in our cohort, the corresponding subphenotypes could be adjudicated on admission (day 0 or 1). In this subset, of the patients with the hypoinflammatory subphenotype at ICU discharge (n = 1227), 1034 (84%) were also adjudicated to the hypoinflammatory subphenotype on ICU admission. In total, 193 patients (16%) changed from the hyperinflammatory at ICU admission to the hypoinflammatory subphenotype at ICU discharge. Of the patients with the hyperinflammatory subphenotype at ICU discharge (n = 83), 76 (92%) were also adjudicated to the hyperinflammatory subphenotype at ICU admission and only 6 (7%) came from the hypoinflammatory subphenotype at ICU admission (Fig. 2).

### Outcome measurements

**Fig. 3:**
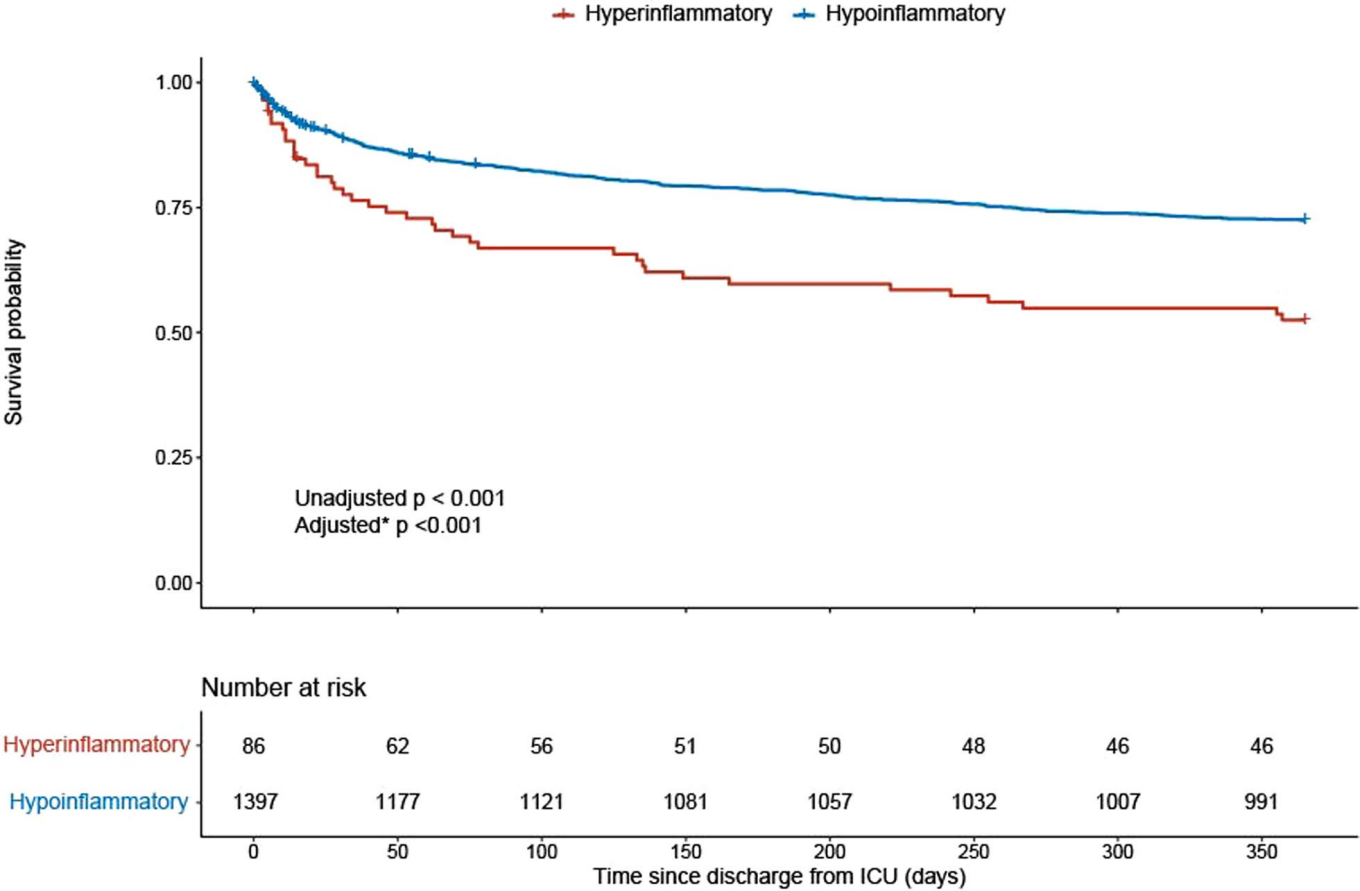
One-year survival curves at ICU discharge according to inflammatory subphenotypes. The log-rank test between the survival curves of the two subphenotypes at ICU discharge showed a *p* < 0.001. Adjusted for age, Charlson comorbidity score, chronic cardiovascular insufficiency, chronic heart failure, chronic kidney disease, malignancy, systolic blood pressure, temperature, platelets, white blood cell count and length of ICU stay at ICU discharge [27] the one year survival was still significant, *p* < 0.001. *ICU* intensive care unit

Mortality was significantly higher in ICU-survivors at ICU discharge adjudicated to the hyperinflammatory subphenotype compared to the hypoinflammatory subphenotype (30-day mortality 21% vs. 11%, *p* = 0.005; one-year mortality 48% vs. 28%, *p* < 0.001, Table 2 and Fig. 3). In a Cox proportional hazards model, adjusted for 11 variables [27], membership to the hyperinflammatory subphenotype at ICU discharge was independently associated with one-year mortality (adjusted hazard ratio (HR) = 2.02 (95% CI 1.37–2.96); *p* < 0.001). A similar result was found when, as a sensitivity analysis, the Cox proportional hazards model was expanded with complications upon ICU admission, admission diagnosis and admission type (HR = 2.10 (95% CI 1.41–3.13); p < 0.001). In this model age, Charlson comorbidity index, chronic cardiovascular insufficiency, malignancy, platelets at ICU discharge, the diagnosis on admission ‘other’ and the admission type ‘surgical’ were all significant associated with one-year mortality. For more information on the model see Additional file 1: Supplementary Table 3. In the hyperinflammatory subphenotype the ICU readmission rate during the remaining study duration of MARS between January 2011 and January 2014 (36% vs. 22%, *p* = 0.006) was significantly higher compared to the hypoinflammatory subphenotype (Table 2). In contrast, the length of ICU stay was shorter in the hyperinflammatory subphenotype (2 vs. 4 days, *p* < 0.001). ICU readmission within the same hospital admission was 24% in the hyperinflammatory vs. 16% in the hypoinflammatory subphenotype (*p* = 0.054). The differences in host response biomarkers between the inflammatory subphenotypes at ICU discharge can be found in the Additional file 1: Supplementary Results, Supplementary Table 4 and Supplementary Fig. 1.

### Readmitted patients within the same hospital admission

In 158 (65%) of 243 patients who were readmitted to the ICU within the same hospital admission, plasma biomarkers were also measured on their corresponding ICU readmission. These 158 patients who were readmitted to the ICU had a SOFA score of 4 [IQR 3–7] at ICU discharge, had a median age of 63 [IQR 54–72] years and 65% (n = 103) was male. The median duration between the admission and readmission was 4 days [IQR 2–10].

**Fig. 4:**
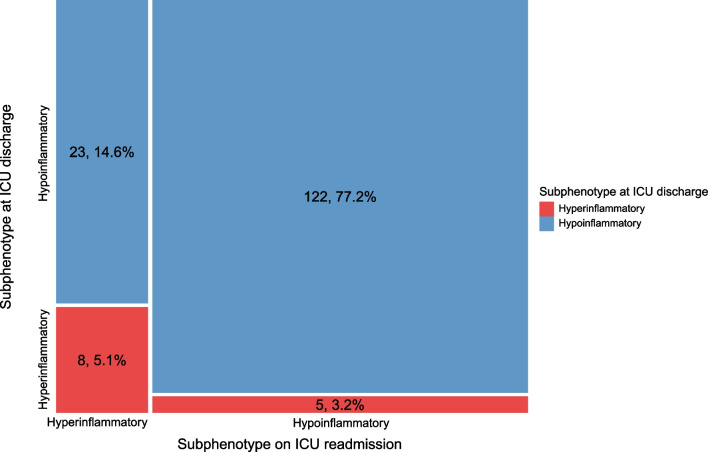
Mosaic plot showing which inflammatory subphenotypes patients had at ICU discharge and on ICU readmission. Mosaic plot showing the allocation of the subphenotypes at ICU discharge and on ICU readmission of the 158 (65%) of the 243 who were readmitted to the ICU and of whom biomarkers were also measured on their first ICU readmission within the same hospital admission. Patients with the hyperinflammatory subphenotype at ICU discharge are depicted in red, with the hypoinflammatory subphenotype in blue. In the left upper corner the patients are displayed who were hypoinflammatory at ICU discharge and hyperinflammatory on ICU readmission (n = 23, 14.6%), in the left lower corner the patients who were both hyperinflammatory at ICU discharge and on ICU readmission (= 8, 5.1%). In the right upper corner the patients are displayed who were both hypoinflammatory at ICU discharge and on ICU readmission (n = 122, 77.2%), in the right lower corner the patients who were hyperinflammatory at ICU discharge and hypoinflammatory on ICU readmission (= 5, 3.2%). *ICU* intensive care unit

In this subset, of the patients with the hypoinflammatory subphenotype at ICU discharge (n = 145), 122 (84%) were also adjudicated to the hypoinflammatory subphenotype at ICU readmission. 23 (16%) of the patients discharged with the hypoinflammatory subphenotype changed to the hyperinflammatory subphenotype at ICU readmission. Of the patients discharged with the hyperinflammatory subphenotype (n = 13), 8 (62%) were also adjudicated to the hyperinflammatory subphenotype at ICU readmission. 5 (38%) patients discharged as hyperinflammatory changed to the hypoinflammatory subphenotype at ICU readmission (Fig. 4).

### Latent class analysis

**Fig. 5:**
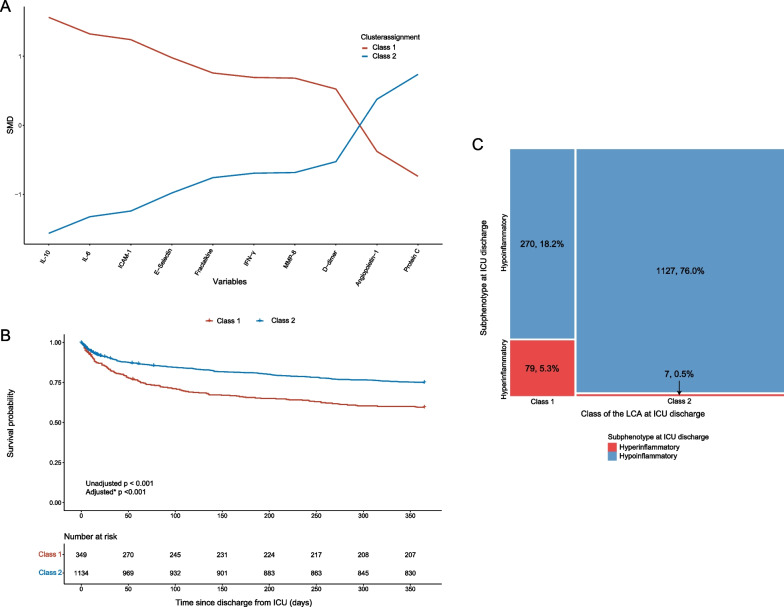
Two subphenotypes identified by the LCA using biomarker data. **A** Profile plot of the two subphenotypes identified by the LCA using biomarker data. All variables used in the latent class analysis are plotted on the x-axis, with the y-axis displaying scales mean values. IL-8 and ang-2/ang-1 were not included due to correlations > 0.6 with other variables, angiopoietin-2 was excluded since one class was completely defined by the values of angiopoietin-2. **B** The log-rank test between the survival curves of the two class at ICU discharge. *Survival was adjusted for age, Charlson comorbidity score, chronic cardiovascular insufficiency, chronic heart failure, chronic kidney disease, malignancy, systolic blood pressure, temperature, platelets, white blood cell count and length of ICU stay at ICU discharge [27]. **C** Mosaic plot showing which patients in the hyperinflammatory and hypoinflammatory belong to which class of the latent class analysis. Patients with the hyperinflammatory subphenotype at ICU discharge are depicted in red, with the hypoinflammatory subphenotype in blue. In the left upper corner the patients are displayed who belonged to the hypoinflammatory subphenotype and class 1 at ICU discharge (n = 270, 18.2%), in the left lower corner the patients who belonged to the hyperinflammatory subphenotype and class 1 (= 79, 5.3%). In the right upper corner the patients are displayed who belonged to the hypoinflammatory subphenotype and class 2 at ICU discharge (n = 1127, 76.0%), in the right lower corner the patients who belonged to the hyperinflammatory subphenotype and class 2 (= 7, 0.5%). *ang* angiopoietin; *ICAM-1* intercellular adhesion molecule-1; *ICU* intensive care unit; *IFN* interferon; *IL* interleukin; *MMP-8* matrix metalloproteinase-8

The following thirteen plasma biomarkers measured at ICU discharge were considered as variables for the LCA: angiopoietin-1, angiopoietin-2, ratio angiopoietin-1/angiopoietin-2, D-dimer, E-Selectin, fractalkine, ICAM-1, INF-γ, IL-6, IL-8, IL-10, MMP-8 and protein C. Three variables, E-Selectin, ICAM-1 and fractalkine, had a low missing percentage (0.2%) before imputation, the other variables used in the LCA were complete. After excluding correlated variables (ratio angiopoietin-1/angiopoietin-2 and IL-8) and excluding angiopoietin-2, see the Additional file 1: Supplementary Results for the rationale, 10 variables remained (Fig. 5A; Additional file 1: Supplementary Fig. 2). Additional file 1: Supplementary Table 5 shows model-fitting statistics for LCA models consisting of 1 to 5 classes. The Vuong-Lo-Mendell-Rubin test showed a *p* < 0.001 for 2, 4 and 5 classes indicating that these class provided an improved fit. The entropy for all the number of classes was 0.75–0.79 and just did not reach the preferable > 0.8. The Bayesian information criterion (BIC) was the lowest for 5 classes, but with 3 or more classes the BIC did not lower significantly and so the increase of the model complexity of adding a third or more classes was considered unnecessary. Therefore, a 2-class latent model was deemed most suitable. The average latent class probability was calculated, which was 90.9% for the whole cohort; 87.8% for class 1 and 94.1% for class 2. The percentage of patients with a probability higher than 90% was 76.1%. In a sensitively analyses, in which the model was run with all five imputation datasets, outcomes and characteristics showed very similar results (data not shown), therefore an imputation dataset was chosen at random.

In the 2-class LCA model 349 (24%) patients were assigned to class 1 and 1134 (76%) to class 4 (Table 3). Class 1 was defined by a more dysregulated inflammatory profile, with the most distinct differences in IL-10 and IL-6 compared to class 2. Furthermore, class 1 was defined by higher endothelial markers, with the most distinct differences in ICAM-1 and E-Selectin. In class 2 patients were significantly older (63 years vs. 61 years, *p* = 0.010) and more often male (63% vs. 55%, *p* = 0.012, Table 3). Patients in class 1 were most severely ill as reflected by higher SOFA scores at ICU discharge (median 6 [IQR 4–8] vs. 3 [IQR 2–5], *p* < 0.001). More differences in medical history, diagnosis and disease severity on admission between patients in the four classes can be found in Additional file 1: Supplementary Table 6. Comparing biomarker levels at ICU-discharge, these results reflected the more dysregulated host response in patients in class 1 compared to patients in class 2, with among others, higher levels of CRP, IL-6, -8, -10, creatinine and lactate (Additional file 1: Supplementary Fig. 3). Mortality was significantly higher in ICU-survivors adjudicated to class 1 compared to class 2 (30-day mortality 17% vs. 10%, *p* = 0.001 and one-year mortality 41% vs. 25%, *p* < 0.001, Table 3 and Fig. 5B). In a Cox proportional hazards model, adjusted for 11 variables [29], membership to class 1 at ICU discharge was independently associated with one-year mortality (adjusted hazard ratio (HR) = 1.86 (95% CI 1.45–2.39); *p* < 0.001). In class 1 the ICU readmission rate was significantly higher (25% vs. 14%, *p* < 0.001) compared to class 2 (Table 3). In contrast, the length of ICU stay was shorter in class 1 (3 vs. 4 days, *p* < 0.001).

**Table 3:** Characteristics and outcomes after ICU discharge, cohort divided based on LCA

	Class 1 n = 349, 24%	Class 2 n = 1134, 76%	*p*-value
*Demographics*			
Age (median [IQR])	61 [49, 70]	63 [51, 71]	0.010
Sex = male (%)	193 (55.3)	714 (63.0)	0.012
Race = White (%)	292 (83.7)	987 (87.0)	0.131
*Disease severity at ICU discharge*			
SOFA score (median [IQR])	6 [4, 8]	3 [2, 5]	< 0.001
*ICU acquired (*≥ *day 2)*			
Shock (%)	16 (4.6)	48 (4.2)	0.887
Acute kidney injury (%)	18 (5.2)	82 (7.2)	0.219
ARDS (%)	12 (16.4)	51 (18.1)	0.876
Sepsis (%)	28 (8.0)	110 (9.7)	0.392
*Restrictions at ICU discharge*			
Restrictions on care (%)			0.663
No restrictions	307 (88.2)	996 (87.9)	
Do not resuscitate	17 (4.9)	70 (6.2)	
Extended restrictions	21 (6.0)	55 (4.9)	
Palliative care	3 (0.9)	12 (1.1)	
*Outcomes*			
LOS hospital (median [IQR])	20 [10, 43]	16 [8, 32]	0.002
LOS ICU (median [IQR])	3 [1, 5]	4 [2, 7]	< 0.001
Hospital mortality (%)	68 (19.5)	109 (9.6)	< 0.001
Days untill mortality (median [IQR])	45 [13–110]	50 [12–172]	0.247
30-day mortality (%)	58 (16.8)	109 (9.8)	0.001
90-day mortality (%)	97 (28.1)	167 (15.1)	< 0.001
1-year mortality (%)	140 (40.6)	278 (25.2)	< 0.001
ICU readmission within the same hospital admission (%)	87 (24.9)	156 (13.8)	< 0.001
ICU readmission in total (%)*	111 (31.8)	235 (20.7)	< 0.001

*ICU readmissions during the complete duration of the MARS study

See Additional file 1: Supplementary Table 6 for information of comorbidities, admission type and diagnoses and disease severity on ICU admission

*ARDS* acute respiratory distress syndrome; *ICU* intensive care unit; *LOS* length of stay; *SOFA* sequential organ failure assessment

When assessing phenotype concordance; patients in the hyperinflammatory subphenotype almost completely belonged to class 1 (n = 79, 92%), only 7 (8%) belonged to class 2. Patients in the hypoinflammatory subphenotype most often belonged to class 2 (n = 1127, 81%), 270 (19%) belonged to class 1 (Fig. 5C), demonstrating a similar pattern in subphenotype adjudication with the largest differences being the distribution of the patients (6% in the hyperinflammatory subphenotype vs. 24% in class 1; 94% in the hyperinflammatory subphenotype vs. 76% in class 2).

## Discussion

This study is the first to demonstrate that the inflammatory subphenotypes previously identified among ARDS patients at ICU discharge are associated with differences in short and long-term outcome. ICU survivors adjudicated to these inflammatory subphenotypes at ICU discharge had an increased mortality. Patients in this hyperinflammatory subphenotype and in the LCA class with the more dysregulated inflammatory profile (class 1) had more derailed markers of coagulation activation, endothelial cell activation and inflammation at ICU discharge.

These findings are in line with previous studies showing that persistent hyperinflammation at either ICU or hospital discharge in ICU survivors is associated with poor physical recovery [31] and higher mortality after hospital and ICU discharge in sepsis-survivors [32, 33]. Furthermore, patients in the hyperinflammatory subphenotype were discharged with significantly higher creatinine levels, which is in line with an earlier study demonstrating that elevated kidney biomarkers measured at ICU discharge in the general ICU population were associated with a poor 1-year outcome [34]. Persistent hyperinflammation at ICU discharge, as found in this study, or even after hospital discharge, plays a detrimental role in outcome [35]. Increased signs of inflammation, measured by CRP, is associated with poor physical recovery during the first 3 months post-ICU discharge [31]. Higher levels of CRP during rehabilitation after hospital admission was furthermore associated with institutionalization and mortality in geriatric patients [36]. Inflammation is a risk factor for physiologic alterations involved in persistent ICU-acquired weakness [37] and might play a role in the development of cognitive disorders by inducing neurotoxicity, endothelial injury and blood–brain barrier dysfunction [38]. The underlying mechanisms linking the acute critical illness to long lasting impairment are however not yet completely understood.

Long lasting impairment after acute critical illness is a frequently occurring health-problem. In a prospective multicenter cohort study conducted in 2345 patients, 50% was suffering from new physical, mental, and/or cognitive health problems 1 year after ICU admission; with 49% having physical impairment, 14% mental impairment, and 4% cognitive impairment [39]. Moreover, the general ICU population has an increased mortality risk after hospital discharge when compared to age and sex-matched population controls, with a mortality rate of 27.5% 3 years after hospital discharge versus 8.2% at 3 years for the general population [40]. There is limited beneficial effect of clinical studies investigating physical rehabilitation [35]. Interventions studied, such as home-based rehabilitation and follow-up clinics, did not yet improve outcome, which raises the question whether the target populations of the studied interventions were appropriate [35]. Using subphenotyping as an enrichment strategy could enhance the likelihood of detecting prevention and therapeutic strategies for specific groups of ICU survivors. Identification of ICU-survivors most at risk for adverse outcomes, could provide information on treatable traits that associate with readmission or death in this group of patients [41]. As an example of a targeted therapeutic strategy, in patients discharged alive from the ICU after experiencing AKI, angiotensin-converting enzyme inhibitor or receptor blocker use after discharge is associated with increased survival [42].

Notably, patients within the hyperinflammatory subphenotype as well as patients within the LCA class 1 with the more dysregulated inflammatory profile were discharged from the ICU significantly quicker, which could not be explained by patients who died during ICU stay since they were excluded from the analysis. This finding seems unrelated to clustering methods as in both techniques the patients with the most derailed host response biomarkers were discharged from the ICU more rapidly compared to the other group. A striking finding, since in other studies a longer ICU stay was associated with higher one-year mortality [27, 43]. The explanation for this shorter length of ICU-stay in patients who were discharged from the ICU with the hyperinflammatory subphenotype, could be related to their trajectory of recovery, i.e. that these patients were discharged to the ward prior to reaching the hypoinflammatory subphenotype. This could also be underlined by the finding of the more derailed host response at ICU discharge and the higher ICU readmission rated in the hyperinflammatory subphenotype. Patients admitted on the ward are less intensively monitored than patients admitted on the ICU, which could make them more susceptible for adverse outcomes. Investigating the value of subphenotyping for assessing the best moment for ICU discharge of individual patients has not been evaluated yet.

A strength of this study is the detailed data collected at ICU discharge including the availability of biomarkers reflective of coagulation activation, endothelial cell activation and inflammation, and the follow-up period of one-year. However, there are several limitations that should be taken into account. First, there might be selection bias in this cohort, as plasma biomarkers were only measured in patients with an infection likelihood of probable or definite, ARDS and matching non-infectious populations, therefore the incidence of specific disease etiologies like sepsis and ARDS might not be representative for the whole ICU population; the mortality might be higher in this cohort compared to a unselected ICU population. Second, the study was not externally validated in another cohort. Detailed information at ICU discharge is often lacking in other datasets, therefore external validation was not possible. Third, since this study has an observational design, any firm conclusions on casual relationships between subphenotypes and one-year mortality cannot be drawn. Last, the LCA model did not reach an entropy of 0.8, which is probably explained by the fact that the class separation at ICU discharge is not as clear as it would have been upon ICU admission. This could be expected, since patients assessed as ready for ICU discharge are probably a more homogenous group than compared to ICU admission.

## Conclusion

In conclusion, this study shows an association between the hyperinflammatory subphenotype and LCA class 1 with the more dysregulated inflammatory profile upon ICU discharge and increased mortality during the subsequent year. This might be linked to the more derailed host response that was found in the hyperinflammatory subphenotype and the LCA class 1 at ICU discharge. The use of subphenotyping as an enrichment strategy could enhance the likelihood of detecting prevention and therapeutic strategies for the ICU survivors most at risk for a poor outcome.

### Supplementary Information

**Additional file 1.** **Supplementary methods.** Supplementary methods on patient selection, comorbidities, complications, biomarker measurements and imputation. **Supplementary results.** Supplementary results on host response biomarkers and latent class analysis. **Supplementary Table 1.** Most common admission diagnoses per admission diagnosis group. **Supplementary Table 2.** Distribution of moment of sampling per inflammatory subphenotype. **Supplementary Table 3.** Results of the adjusted Cox proportional hazards model for associations with one-year mortality. **Supplementary Table 4.** Comparison of biomarkers in the complement-, endothelial and inflammatory pathways at ICU discharge between the hyperinflammatory and hypoinflammatory subphenotype. **Supplementary Table 5.** Model-fit statistics for different number of latent classes. **Supplementary Table 6.** Characteristics at ICU admission, cohort divided based on LCA. **Supplementary Fig. 1.** Biomarker differences at ICU discharge according to inflammatory subphenotypes. **Supplementary Fig. 2.** Correlation plot with variables intended to use in LCA. **Supplementary Fig. 3.** Biomarker plots stratified per LCA class.

## Data Availability

The data of our cohort (MARS) is available at the Gene Expression Omnibus public repository of NCBI under accession number GSE65682.
