# Supplementary material for: Inflammatory subphenotypes previously identified in ARDS are associated with mortality at intensive care unit discharge: a secondary analysis of a prospective observational study
Source: Crit Care. 2024 May 7;28:151. doi: 10.1186/s13054-024-04929-9 (PMC11077885; doi:10.1186/s13054-024-04929-9)
Supplement: Supplementary file 1 — Additional file 1. Supplementary methods. Supplementary methods on patient selection, comorbidities, complications, biomarker measurements and imputation. Supplementary results. Supplementary results on host response biomarkers and latent class analysis. Supplementary Table 1. Most common admission diagnoses per admission diagnosis group. Supplementary Table 2. Distribution of moment of sampling per inflammatory subphenotype. Supplementary Table 3. Results of the adjusted Cox proportional hazards model for associations with one-year mortality. Supplementary Table 4. Comparison of biomarkers in the complement-, endothelial and inflammatory pathways at ICU discharge between the hyperinflammatory and hypoinflammatory subphenotype. Supplementary Table 5. Model-fit statistics for different number of latent classes. Supplementary Table 6. Characteristics at ICU admission, cohort divided based on LCA. Supplementary Fig. 1. Biomarker differences at ICU discharge according to inflammatory subphenotypes. Supplementary Fig. 2. Correlation plot with variables intended to use in LCA. Supplementary Fig. 3. Biomarker plots stratified per LCA class. [file 13054_2024_4929_MOESM1_ESM.docx]

**Supplement**

**Inflammatory subphenotypes previously identified in ARDS are associated with mortality at intensive care unit discharge: a secondary analysis of a prospective observational study**

Marleen A. Slim, MD, Rombout B.E. van Amstel, MD, Lieuwe D.J. Bos, MD, PhD, Olaf L. Cremer, MD, PhD, MARS Consortium, W. Joost Wiersinga, MD, PhD, Tom van der Poll, MD, PhD, Lonneke A. van Vught, MD, PhD

| **Content** | **Page** |
| --- | --- |
| Supplementary methods | 2 |
| Supplementary results | 3 |
| Supplementary Table 1. Most common admission diagnoses per admission diagnosis group | 5 |
| Supplementary Table 2. Distribution of moment of sampling per inflammatory subphenotype | 6 |
| Supplementary Table 3. Results of the adjusted Cox proportional hazards model for associations with one-year mortality | 7 |
| Supplementary Table 4. Comparison of biomarkers in the complement-, endothelial and inflammatory pathways at ICU discharge between the hyperinflammatory and hypoinflammatory subphenotype | 8 |
| Supplementary Table 5. Model-fit statistics for different number of latent classes | 9 |
| Supplementary Table 6. Characteristics at ICU admission, cohort divided based on LCA | 10 |
| Supplementary Figure 1. Biomarker differences at ICU discharge according to inflammatory subphenotypes | 11 |
| Supplementary Figure 2. Correlation plot with variables intended to use in LCA | 12 |
| Supplementary Figure 3. Biomarker plots stratified per LCA class | 13 |

**Supplementary methods**

*Patient selection*

For this manuscript we included all consecutive patients in the MARS of whom biomarkers were measured at ICU discharge. The selection for this cohort was done for previous papers (1-7), the selection was done as following:

- All patients with a probable or possible infectious diagnoses within 48hours after ICU admission (sepsis on admission), or during ICU stat (ICU-acquired sepsis) accompanied by at least one additional variable as described in the 2001 International Sepsis Definitions (8) (defined patients admitted for sepsis);
- All patients defined as noninfectious controls (for abdominal sepsis patients these were patients who underwent abdominal surgery for a noninfectious condition, for community acquired pneumonia (CAP) these were patients with a *post hoc* CAP likelihood of “none”, for hospital acquired pneumonia (HAP) these were also patients with a post hoc HAP likelihood of “none” and so forth);
- All patients with ARDS or AKI on ICU admission or during ICU stay;
- All patients with medical or surgical shock.

*Comorbidities*

Comorbidities were defined as described previously (3), to know; cardiovascular insufficiency was defined as having a medical history of congestive heart failure, myocardial infarction, chronic cardiovascular disease, peripheral vascular disease or cerebrovascular disease. Immunocompromise was defined as a medical history of immune deficiency, human immune deficiency virus (HIV) or acquired immune deficiency syndrome (AIDS), asplenia, hematological malignancy or by the use of corticosteroids or antineoplastic medication. Malignancy was defined as a medical history of either non-metastatic solid tumor, metastatic malignancy or hematologic malignancy. Renal insufficiency was defined as a history of chronic renal insufficiency or chronic intermitted hemodialysis or continuous ambulatory peritoneal dialysis. Respiratory insufficiency was defined as chronic obstructive pulmonary disease, respiratory insufficiency in the medical history or home oxygen use/ventilator support.

*Complications*

Complications on the day of admission were defined as events that were present on the day of intensive care unit (ICU) admission or one day thereafter; ICU acquired complications as events that started 2 days or more after ICU admission. Sepsis was defined as the presence of an infection diagnosed within 24 hours after ICU admission with a probable or definite likelihood (9) accompanied by a SOFA score≥ 2 according to the sepsis-3 criteria (10). Shock was defined as the use of noradrenaline for hypotension in a dose of >0.1 µg/kg/min during at least 50% of day. Acute respiratory distress syndrome (ARDS) and acute kidney injury (AKI) were assessed using strict preset criteria (11, 12).

*Biomarker measurements*

Whole blood samples were centrifuged directly (1500 G for 15 min) and frozen at -80°C within 4 hours after blood draw. Interleukin-6 (IL-6), IL-8, IL-10, interferon-gamma, fractalkine, soluble intercellular adhesion molecule-1 (ICAM-1), and soluble E-selectin were measured by FlexSet cytometric bead array (BD Biosciences, San Jose, CA) using FACS Calibur (Becton Dickinson, Franklin Lakes, NJ). Matrix metalloproteinase (MMP)-8, angiopoietin-1, angiopoietin-2, protein C (all R&D Systems, Abingdon, UK), and D-dimer (Procartaplex; eBioscience, San Diego, CA) were measured by Luminex multiplex assay using BioPlex 200 (BioRad, Hercules, CA) (2).

*Imputation*

Missing biomarker values on ICU discharge used in the latent class analysis (LCA) were imputed using multivariate imputation by chained equations algorithm with predictive mean matching with estimates combined of five imputed datasets with 20 iterations each (13). All other data including clinical and outcome data regarding survival and complication rates were not imputed.

**Supplementary results**

*Host response biomarkers*

We compared 17 host response plasma protein biomarkers between the subphenotypes at ICU discharge, of which 14 showed significant differences between the two groups (Supplementary Figure 1; Supplementary Table 3). Biomarker levels across all pathophysiological domains differed between subphenotypes at ICU discharge, with stronger coagulation activation, endothelial cell activation and inflammation in the hyperinflammatory subphenotype (Supplementary Figure 1).

*Latent class analysis*

The decision to exclude angiopoietin-2 was based on the following findings. If we would have included angiopoietin-2 in the LCA, 3 classes would have been the best fit. In one of the three classes, consisting of 90 patients, all patients had very low concentrations of angiopoietin-2. Out of the 90 patients in this class, 86 patients had an unmeasurable low angiopoietin-2 concentration, which was set to the lower limit of quantification (1.8 pg/ml). The 4 remaining patients in this class had concentrations between 7.4 and 17.9. In contrast, of all the 1483 included in our cohort the median concentration was 3830.0 pg/ml [IQR 1922.0-7458.9]. Furthermore, according to the ‘Practitioner’s Guide to Latent Class Analysis’ (14), we must consider rejecting this model for the following reasons: the number of patients in the class is <10% of the sample size, which suggests a poor fitting model, and a single indicator is the pre-dominant determinant of one of the classes. Moreover, there was no clear evidence that there is a biological substrate explaining this class. In this class, all other markers for endothelial (dys)function, which are angiopoietin-1, E-Selectin, fractalkine and ICAM-1, have similar concentrations to another class. This means that all other endothelial biomarkers do not follow the same (extreme) pattern of angiopoietin-2. Taken together, we concluded that based on the fact that angiopoietin-2 is responsible as a single marker for one class, which was driven by concentrations of angiopoietin -2 below the lower limit of detection and Practioner’s Guide to Latent Class Analysis’ suggests to reject this model based on sample size, the best decision would be to exclude angiopoietin-2 from our LCA.

**Supplementary Table 1. Most common admission diagnoses per admission diagnosis group**

1. Non-infectious patients

| **Admission diagnosis group** | **APACHE IV admission diagnosis** | **Frequency (n)** |
| --- | --- | --- |
| **Cardiovascular** | Cardiac arrest | 53 |
|  | CABG alone, coronary artery bypass grafting | 35 |
|  | Aneurysm, abdominal aortic | 16 |
|  | CABG with aortic valve replacement | 14 |
|  | Infarction, acute myocardial | 12 |
|  | Congestive heart failure | 11 |
|  | CABG with mitral valve repair | 8 |
|  | Shock, cardiogenic | 7 |
|  | Aneurysm, abdominal aortic; with rupture | 6 |
|  | Rhythm disturbance (ventricular) | 6 |
|  | Aortic valve replacement (isolated) | 5 |
| **Gastrointestinal** | Cancer-other GI tract, surgery for | 21 |
|  | Cancer-stomach, surgery for | 10 |
|  | GI surgery, other | 10 |
|  | Whipple-surgery for pancreatic cancer | 8 |
|  | Cancer-colon/rectal, surgery for | 6 |
|  | GI Obstruction, surgery for | 5 |
|  | GI Perforation/rupture, surgery for | 5 |
| **Metabolic** | Overdose, other toxin, poison or drug | 3 |
| **Neurological** | Hematoma, subdural, surgery for | 12 |
|  | Hemorrhage/hematoma, intracranial | 12 |
|  | Subarachnoid hemorrhage/intracranial aneurysm | 10 |
|  | Hemorrhage/hematoma-intracranial, surgery for | 6 |
|  | Cerebrovascular accident/stroke | 5 |
| **Other** | Heart transplant | 3 |
|  | Thoracotomy for esophageal cancer | 3 |
| **Respiratory** | Pneumonia, bacterial | 11 |
|  | Embolus, pulmonary | 7 |
|  | Pneumonia, aspiration | 6 |
|  | Respiratory- medical, other | 6 |
| **Trauma** | Head (CNS) only trauma | 4 |

Admissions with a frequency of at least 5 were included in this table, except for the groups metabolic, other and trauma because of the low frequencies of diagnoses in these groups. These diagnoses on admission are based on the APACHE IV admission diagnoses. Abbreviations: CABG, Coronary artery bypass graft surgery; CNS, central nervous system; GI, gastrointestinal.

1. Sepsis patients

| **Source of infection** | **Frequency of sepsis patients (n)** |
| --- | --- |
| **Cardiovascular** | 97 |
| **Gastrointestinal** | 184 |
| **Metabolic** | 2 |
| **Neurological** | 27 |
| **Other** | 158 |
| **Respiratory** | 459 |

**Supplementary Table 2. Distribution of moment of sampling per inflammatory subphenotype**

| **Sample day** | **Hyperinflammatory**  **n = 86** | **Hypoinflammatory**  **n = 1397** |
| --- | --- | --- |
| Day of ICU discharge | 50 (58%) | 909 (65%) |
| Day before ICU discharge (-1) | 34 (40%) | 385 (28%) |
| Two days before ICU discharge (-2) | 2 (2%) | 103 (7%) |

Abbreviations: ICU, intensive care unit

| **Variable** | **Hazard ratio** | **95% CI** | **P value** |
| --- | --- | --- | --- |
| Subphenotype – hyperinflammatory | 2.10 | 1.41 – 3.13 | < 0.001 |
| Age | 1.02 | 1.01 – 1.04 | < 0.001 |
| Charlson comorbidity index | 1.09 | 1.01 – 1.17 | 0.024 |
| Chronic cardiovascular insufficiency | 1.73 | 1.05 – 2.86 | 0.032 |
| Chronic heart failure | 0.82 | 0.52 – 1.31 | 0.414 |
| Chronic kidney disease | 1.13 | 0.76 – 1.68 | 0.533 |
| Malignancy | 1.57 | 1.13 – 2.19 | 0.008 |
| Systolic blood pressure* | 1.00 | 0.99 – 1.00 | 0.356 |
| Temperature* | 0.95 | 0.86 – 1.06 | 0.347 |
| Platelets* | 1.00 | 1.00 – 1.00 | 0.007 |
| White blood cell count* | 1.00 | 0.98 – 1.02 | 0.919 |
| Length of ICU stay | 1.01 | 0.99 – 1.02 | 0.347 |
| AKI on admission | 0.86 | 0.67 – 1.11 | 0.245 |
| ARDS on admission | 1.20 | 0.89 – 1.63 | 0.237 |
| Sepsis on admission | 1.25 | 0.86 – 1.80 | 0.242 |
| Diagnosis on admission - gastrointestinal | 0.83 | 0.57 – 1.20 | 0.316 |
| Diagnosis on admission - Metabolic | 0.59 | 1.14 – 2.48 | 0.473 |
| Diagnosis on admission - Neurological | 1.32 | 0.79 – 2.20 | 0.291 |
| Diagnosis on admission - Other | 0.50 | 0.32 – 0.79 | 0.002 |
| Diagnosis on admission - Respiratory | 0.90 | 0.63 – 1.28 | 0.557 |
| Diagnosis on admission - Trauma | 0.22 | 0.03 – 1.64 | 0.142 |
| Admission type – Surgical | 0.56 | 0.36 – 0.87 | 0.011 |

**Supplementary Table 3. Results of the adjusted Cox proportional hazards model for associations with one-year mortality**

*At ICU discharge. Abbreviations: AKI, acute kidney injury; ARDS, acute respiratory distress syndrome; CI, confidence interval; ICU, intensive care unit.

**Supplementary Table 4. Comparison of biomarkers in coagulation, endothelial and inflammatory pathways at ICU discharge between the hyperinflammatory and hypoinflammatory subphenotype**

| **Biomarker,**  **median [IQR]** | **Hyperinflammatory**  **(n = 86)** | **Hypoinflammatory**  **(n = 1397)** | **p-value** | **Effect size (Hedges’ g)** | **95% CI** |
| --- | --- | --- | --- | --- | --- |
| **Coagulation** | | | | | |
| D-Dimer (ng/ml) | 11842.50 [5707.78-21406.75] | 7473.10 [3558.90-14397.00] | <0.001 | 0,450256245 | (0,23 - 0,7) |
| Platelets (pg/ml) | 143.00 [61.50-229.50] | 204.00 [139.00-301.00] | <0.001 | 0,507643431 | (-0,75 - -0,27) |
| PT (pg/ml) | 15.75 [13.65-18.95] | 13.10 [11.50-15.10] | <0.001 | 0,476881305 | (0,35 - 0,63) |
| **Endothelial cell activation** | | | | | |
| Angiopoietin-1 (pg/ml) | 1058.21 [409.03-3627.87] | 2073.77 [835.31-5229.35] | 0.001 | -0,213605021 | (-0,48 - 0,01) |
| Angiopoietin-2 (pg/ml) | 9291.40 [4926.61-21848.95] | 3670.16 [1858.86-7089.99] | <0.001 | 0,697927425 | (0,53 - 0,92) |
| Ang-2/Ang-1 (pg/ml) | 8.67 [1.97-41.43] | 1.59 [0.48-5.88] | <0.001 | -0,041568284 | (-0,08 - 0,2) |
| E-Selectin (pg/ml) | 49.33 [16.28-99.10] | 23.59 [12.73-45.81] | <0.001 | 0,513655116 | (0,33 - 0,68) |
| Fractalkine (pg/ml) | 32.93 [17.55-82.94] | 17.90 [12.75-32.60] | <0.001 | 0,321231851 | (0,23 - 0,48) |
| ICAM-1 (ng/ml) | 298.59 [168.86-487.12] | 147.38 [89.21-245.23] | <0.001 | 0,881614783 | (0,68 - 1,1) |
| **Inflammation** | | | | | |
| CRP (pg/ml) | 180.00 [117.50-253.50] | 86.00 [42.00-162.00] | <0.001 | 0,813300477 | (0,54 - 1,11) |
| IL-6 (pg/ml) | 234.36 [75.76-1322.74] | 23.86 [9.87-57.83] | <0.001 | 0,271365435 | (0,12 - 0,42) |
| IL-10 (pg/ml) | 21.49 [9.12-75.28] | 3.36 [1.67-7.10] | <0.001 | 0,341414195 | (0,28 - 0,91) |
| IFN- γ (pg/ml) | 13.12 [3.90-84.58] | 5.10 [1.68-12.23] | <0.001 | 0,440636191 | (0,34 - 0,58) |
| MMP-8 (pg/ml) | 3551.15 [858.57-12387.31] | 788.07 [357.57-1907.96] | <0.001 | 0,654052051 | (0,52 - 0,88) |
| WBC (pg/ml) | 13.25 [8.30-19.92] | 11.80 [8.90-15.90] | 0.249 | 0,200167018 | (-0,07 - 0,45) |
| **Other** | | | | | |
| Creatinine (pg/ml) | 115.00 [76.00-189.00] | 76.00 [56.00-118.00] | <0.001 | 0,407910646 | (0,21 - 0,57) |
| Lactate (pg/ml) | 2.20 [1.40-2.80] | 1.60 [1.10-2.40] | 0.003 | 0,369179957 | (0,1 - 0,61) |

Biomarkers in the complement-, endothelial and inflammatory pathways at ICU discharge expressed as median concentrations compared using a Mann-Whitney U test and with Hedges’ g to demonstrate the effect size. Abbreviations: ang: angiopoietin; CI, confidence interval; CRP, C-reactive protein; ICAM-1, intercellular adhesion molecule-1; ICU, intensive care unit; IFN, interferon; IL, interleukin; MMP8, matrix metalloproteinase-8; PT, prothrombin time; WBC, white blood cell count.

**Supplementary Table 5. Model-fit statistics for different number of latent classes.**

| **Classes** | **Number of patients per class** | **BIC** | ***p*-value*** | **Entropy** |
| --- | --- | --- | --- | --- |
| 1 | 1483 | 42221.75 | - | - |
| 2 | 1134, 349 | 41242.91 | <0.001 | 0,75 |
| 3 | 919, 523, 41 | 40821.92 | 0.23 | 0,78 |
| 4 | 761, 377, 295, 50 | 40708.16 | 1.00 | 0,75 |
| 5 | 733, 366, 300, 43, 41 | 40025.18 | 1.00 | 0,79 |

*By Vuong-Lo-Mendell-Rubin test.

Abbreviations: BIC, Bayesian information criterion; LMR-LRT, Lo–Mendell–Rubin adjusted likelihood ratio test.

**Supplementary Table 6. Characteristics at ICU admission, cohort divided based on LCA**

|  | **Class 1**  **n = 349, 24%** | **Class 2**  **n = 1134, 76%** | ***p*-value** |
| --- | --- | --- | --- |
|  |  |  |  |
| ***Medical history*** |  |  |  |
| No comorbidities (%) | 86 (24.6) | 347 (30.6) | 0.038 |
| Cardiovascular compromise (%) | 100 (28.7) | 340 (30.0) | 0.683 |
| COPD (%) | 39 (11.2) | 137 (12.1) | 0.716 |
| Diabetes (%) | 70 (20.1) | 218 (19.2) | 0.790 |
| Hypertension (%) | 94 (26.9) | 344 (30.3) | 0.250 |
| Immunocompromise (%) | 93 (26.6) | 202 (17.9) | <0.001 |
| Malignancy (%) | 79 (22.6) | 165 (14.6) | 0.001 |
| Renal insufficiency (%) | 58 (16.6) | 102 (9.0) | <0.001 |
| Respiratory insufficiency (%) | 51 (14.6) | 188 (16.6) | 0.430 |
| Charlson comorbidity score (median [IQR]) | 3 [2, 5] | 3 [1, 5] | 0.389 |
|  |  |  |  |
| ***Diagnosis on admission*** |  |  |  |
| Surgical admission (%) | 46 (13.2) | 242 (21.3) | 0.001 |
| Diagnosis on admission (%) |  |  | <0.001 |
| Cardiovascular | 61 (17.5) | 262 (23.1) |  |
| Gastrointestinal | 106 (30.4) | 176 (15.5) |  |
| Metabolic | 3 (0.9) | 11 (1.0) |  |
| Neurological | 12 (3.4) | 88 (7.8) |  |
| Other | 81 (23.2) | 125 (11.0) |  |
| Respiratory | 82 (23.5) | 432 (38.1) |  |
| Trauma | 4 (1.1) | 40 (3.5) |  |
|  |  |  |  |
| ***On admission (day 0 & 1)*** |  |  |  |
| Mechanical ventilation (%) | 233 (66.8) | 955 (84.2) | <0.001 |
| Shock (%) | 90 (25.9) | 289 (25.5) | 0.944 |
| Acute kidney injury (%) | 158 (45.3) | 282 (24.9) | <0.001 |
| ARDS (%) | 61 (17.5) | 231 (20.4) | 0.267 |
| Sepsis (%)  *Of which septic shock* | 270 (77.4)  *71 (20.4)* | 657 (57.9)  *186 (16.4)* | <0.001  *0.100* |
|  |  |  |  |
| ***Disease severity on admission*** |  |  |  |
| APACHE IV Score (median [IQR]) | 74 [59, 95] | 69 [53, 88] | <0.001 |
| SOFA (median [IQR]) | 7 [5, 9] | 6 [4, 8] | <0.001 |

Abbreviations: APACHE, acute physiology and chronic health evaluation; ARDS, acute respiratory distress syndrome; COPD, chronic obstructive pulmonary disease; SOFA, sequential organ failure assessment

**Supplementary Figure 1. Biomarker differences at ICU discharge according to inflammatory subphenotypes**

**
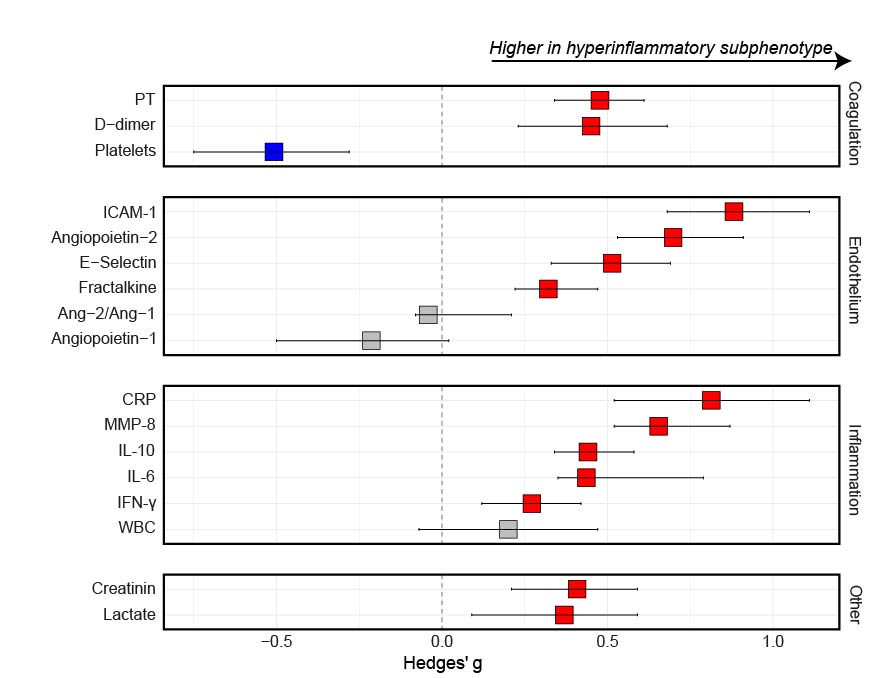
**

The magnitude in biomarker differences is expressed with Hedges’g with their 95% confidence interval. Confidence intervals were obtained from 1000 times bootstrapping. Red indicates higher levels in patients with the hyperinflammatory subphenotype, blue is higher in the hypoinflammatory subphenotype. Abbreviations: ang: angiopoietin; CRP, C-reactive protein; ICAM-1, intercellular adhesion molecule-1; ICU, intensive care unit; IFN, interferon; IL, interleukin; MMP-8, matrix metalloproteinase-8; PT, prothrombin time; WBC, white blood cell count.

**Supplementary Figure 2. Correlation plot with variables intended to use in LCA**

**
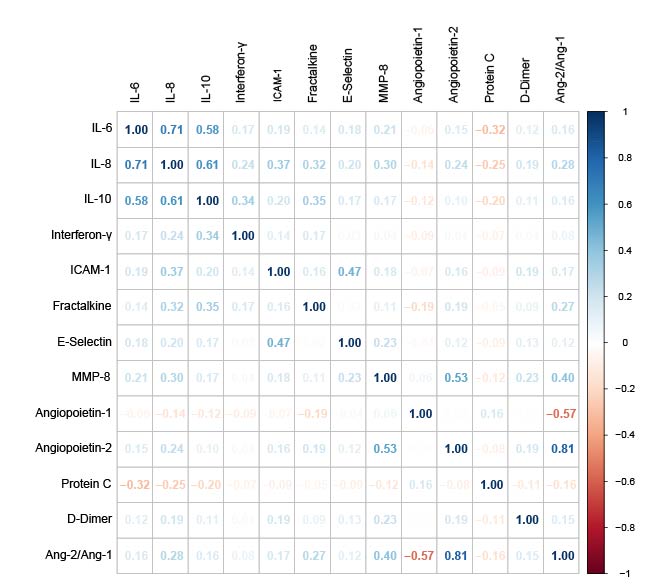
**

Correlation plot of all variables intended to use in LCA. The color represents the correlation coefficient (legend included). Spearman rank-order correlation coefficient was used for calculation. Of variables with correlations >0.6, one of the two was excluded from the analysis; IL-8 and ratio Ang-2/Ang-1 were excluded. Abbreviations: ang: angiopoietin; ICAM-1, intercellular adhesion molecule-1; IFN, interferon; IL, interleukin; MMP-8, matrix metalloproteinase-8.

**Supplementary Figure 3. Biomarker differences at ICU discharge stratified per LCA class**

**
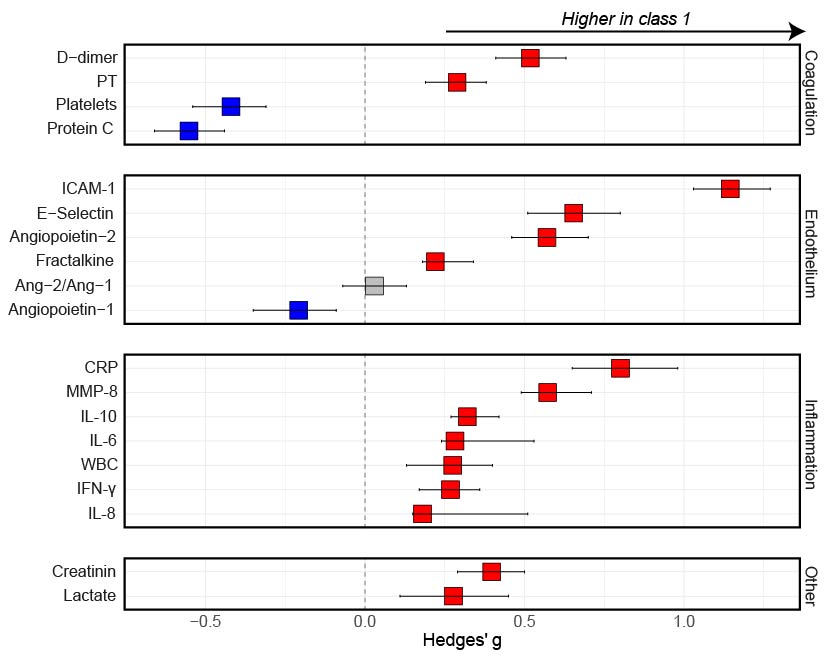
**

The magnitude in biomarker differences is expressed with Hedges’g with their 95% confidence interval. Confidence intervals were obtained from 1000 times bootstrapping. Red indicates higher levels in patients with class 1, blue is higher in class 2. Abbreviations: ang: angiopoietin; CRP, C-reactive protein; ICAM-1, intercellular adhesion molecule-1; IFN, interferon; IL, interleukin; MMP-8, matrix metalloproteinase-8; PT, prothrombin time; WBC, white blood cell count.

**References**

1. van Vught LA, Wiewel MA, Hoogendijk AJ, Frencken JF, Scicluna BP, Klein Klouwenberg PMC, et al. The Host Response in Patients with Sepsis Developing Intensive Care Unit-acquired Secondary Infections. Am J Respir Crit Care Med. 2017;196(4):458-70.

2. van Vught LA, Scicluna BP, Wiewel MA, Hoogendijk AJ, Klein Klouwenberg PMC, Ong DSY, et al. Association of Gender With Outcome and Host Response in Critically Ill Sepsis Patients. Crit Care Med. 2017;45(11):1854-62.

3. van Vught LA, Klein Klouwenberg PM, Spitoni C, Scicluna BP, Wiewel MA, Horn J, et al. Incidence, Risk Factors, and Attributable Mortality of Secondary Infections in the Intensive Care Unit After Admission for Sepsis. Jama. 2016;315(14):1469-79.

4. van Vught LA, Wiewel MA, Klein Klouwenberg PM, Hoogendijk AJ, Scicluna BP, Ong DS, et al. Admission Hyperglycemia in Critically Ill Sepsis Patients: Association With Outcome and Host Response. Crit Care Med. 2016;44(7):1338-46.

5. van Vught LA, Scicluna BP, Wiewel MA, Hoogendijk AJ, Klein Klouwenberg PM, Franitza M, et al. Comparative Analysis of the Host Response to Community-acquired and Hospital-acquired Pneumonia in Critically Ill Patients. Am J Respir Crit Care Med. 2016;194(11):1366-74.

6. van Vught LA, Scicluna BP, Hoogendijk AJ, Wiewel MA, Klein Klouwenberg PM, Cremer OL, et al. Association of diabetes and diabetes treatment with the host response in critically ill sepsis patients. Crit Care. 2016;20(1):252.

7. Claushuis TA, van Vught LA, Scicluna BP, Wiewel MA, Klein Klouwenberg PM, Hoogendijk AJ, et al. Thrombocytopenia is associated with a dysregulated host response in critically ill sepsis patients. Blood. 2016;127(24):3062-72.

8. Levy MM, Fink MP, Marshall JC, Abraham E, Angus D, Cook D, et al. 2001 SCCM/ESICM/ACCP/ATS/SIS International Sepsis Definitions Conference. Crit Care Med. 2003;31(4):1250-6.

9. Klein Klouwenberg PM, Ong DS, Bos LD, de Beer FM, van Hooijdonk RT, Huson MA, et al. Interobserver agreement of Centers for Disease Control and Prevention criteria for classifying infections in critically ill patients. Crit Care Med. 2013;41(10):2373-8.

10. Singer M, Deutschman CS, Seymour CW, Shankar-Hari M, Annane D, Bauer M, et al. The Third International Consensus Definitions for Sepsis and Septic Shock (Sepsis-3). Jama. 2016;315(8):801-10.

11. Bernard GR, Artigas, A., Brigham, K. L., Carlet, J., Falke, K., Hudson, L., Lamy, M., Legall, J. R., Morris, A., & Spragg, R. The American-European Consensus Conference on ARDS. Definitions, mechanisms, relevant outcomes, and clinical trial coordination. American journal of respiratory and critical care medicine. 1994;149(3 Pt 1):818–24.

12. Bellomo R, Ronco C, Kellum JA, Mehta RL, Palevsky P, Acute Dialysis Quality Initiative w. Acute renal failure - definition, outcome measures, animal models, fluid therapy and information technology needs: the Second International Consensus Conference of the Acute Dialysis Quality Initiative (ADQI) Group. Crit Care. 2004;8(4):R204-12.

13. van Buuren S G-OK. mice: Multivariate Imputation by Chained Equations in R. J Stat Softw. 2011;45(3):1–67.

14. Sinha P, Calfee CS, Delucchi KL. Practitioner's Guide to Latent Class Analysis: Methodological Considerations and Common Pitfalls. Crit Care Med. 2021;49(1):e63-e79.
